# Study on Multi-Station Identification Technology of Lightning Electromagnetic Pulses (LEMPs) Based on Deep Learning

**DOI:** 10.3390/s25237217

**Published:** 2025-11-26

**Authors:** Fang Xiao, Qiming Ma, Jiajun Song, Shangbo Yuan, Chaoyi Hu, Jiaquan Wang, Xiao Zhou

**Affiliations:** Institute of Electrical Engineering, Chinese Academy of Sciences, Beijing 100190, China; xiaofang@mail.iee.ac.cn (F.X.); maqiming@mail.iee.ac.cn (Q.M.); songjiajun@mail.iee.ac.cn (J.S.); yuanshangbo@mail.iee.ac.cn (S.Y.); huchaoyi@mail.iee.ac.cn (C.H.)

**Keywords:** lightning, multi-station, deep learning, lightning electromagnetic pulses, convolutional neural network (CNN)

## Abstract

Given the increasing threat of lightning to modern electronic systems and human activities, the accurate identification and classification of lightning electromagnetic pulses has become a critical research focus, prompting the present study. A dataset was established by collecting lightning electromagnetic signals generated by various types of lightning under diverse environmental conditions via the lightning location system of the Institute of Electrical Engineering, Chinese Academy of Sciences. Subsequently, A deep learning model integrating a convolutional neural network was developed for feature extraction and pattern recognition using the multi-station data. Experimental results demonstrate that the proposed model significantly improves LEMP identification accuracy (exceeding 97%) compared to existing single-station methods. Moreover, it effectively uncovers complex hidden features within the data, outperforming conventional approaches in both accuracy and robustness. In conclusion, the proposed deep learning model offers a reliable technical foundation for lightning monitoring and localization based on LEMP signals.

## 1. Introduction

Lightning Electromagnetic Pulses (LEMPs) not only pose risks of system blackouts by damaging power system facilities [1], irreversible damage to communication networks and precision electronic equipment [2], but also easily trigger disasters such as lightning-induced forest fires [3,4], constituting a dual threat to both the ecological environment and human life and property safety. LEMPs are simply classified into cloud-to-ground (CG) and intracloud types based on their occurrence locations. Intracloud lightning is further divided into ordinary intracloud lightning (IC) and Narrow Bipolar Events (NB), with the latter exhibiting stronger radiation intensity and greater hazards. Consequently, achieving efficient and accurate identification of CG, IC, and NB has become a critical issue requiring urgent resolution.

Existing LEMP identification methods mostly rely on empirical formulas or basic feature extraction techniques [5], which have exposed significant limitations in practical applications, such as insufficient classification accuracy and weak environmental adaptability. With the rapid development of deep learning technology, it has provided a new technical approach for the efficient processing of complex electromagnetic signals [6,7]. However, most of the existing research results focus on the development of identification technologies in single-station detection scenarios [8,9,10,11], and have not fully considered the actual engineering requirements of lightning detection: lightning location systems usually achieve accurate positioning based on multi-station collaborative observation [12]. Limited by the signal acquisition range and information dimension of a single observation point, single-station identification technology is difficult to achieve accurate identification of lightning signals in complex electromagnetic environments, and is prone to misjudgment, which in turn adversely affects the accuracy of subsequent positioning results.

The lightning location system is based on multi-station detection technology, and GPS/Beidou provides trivial synchronization. By deploying multiple detection stations and utilizing the Time Difference of Arrival method, it can achieve high-precision positioning of lightning locations [13,14,15]. At the same time, deep learning technology has been widely applied in the field of waveform recognition [16,17], providing a new solution for the classification of LEMPs. Nevertheless, existing methods still face the following scientific problems and technical challenges: (1) signal synchronization and noise suppression in multi-station detection systems; (2) inadequate capability to discriminate between multiple types of LEMP signals in highly complex electromagnetic environments; and (3) insufficient robustness of current algorithms in practical applications that need to be further enhanced.

Targeting the above issues, this study proposes a multi-station identification method for LEMPs based on deep learning. The method establishes a multi-station synchronous observation system [8] to acquire comprehensive, multi-dimensional LEMP data. By leveraging the feature learning and pattern classification capabilities of deep learning algorithms, an efficient multi-station joint recognition model [6,17] is developed. This approach significantly improves the recognition accuracy of LEMP signals across diverse scenarios, which is adaptable to the respective environmental conditions of existing lightning detection stations, thereby providing reliable technical support for subsequent precise localization. Experimental validation demonstrates that the proposed method achieves substantially higher accuracy and broader classification capability compared to conventional single-station approaches, while maintaining robust performance in field applications.

## 2. Materials and Methods

### 2.1. Data

The lightning data employed in this study were obtained from the ADTD lightning detection network operated by the Institute of Electrical Engineering, Chinese Academy of Sciences (IEECAS). The ADTD lightning detection network comprises over 500 stations distributed across China and the Asia–Pacific region [6,18], which was initially deployed in 2013 and underwent a significant upgrade in 2018, transitioning to advanced waveform acquisition systems. In 2019, IEECAS deployed approximately 70 additional lightning detection stations across Belt and Road countries, including Cambodia, Sri Lanka, Laos, and Pakistan, thereby enhancing the environmental diversity of electromagnetic pulse data.

Each lightning detection station is equipped with dual-mode GPS/BeiDou satellite positioning and timing units to ensure temporal synchronization. The channel bandwidth of the station is 3 kHz to 400 kHz. The LEMPs employed in the experiments were sampled at 1 MSPS (Mega Samples Per Second), with each signal record comprising 1000 data points over a 1-millisecond duration.

**Figure 1:**
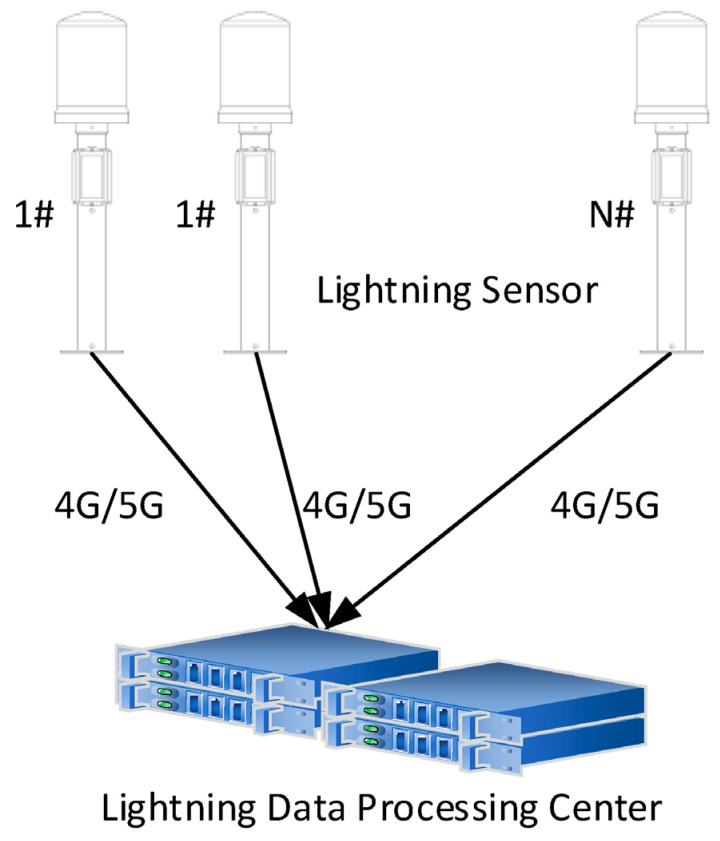
The data processing of the ADTD lightning detection network.

Figure 1 illustrates the acquisition, transmission, and processing workflow of the ADTD lightning detection network. All the lightning detection stations transmit the acquired waveform data in real time to the Lightning Data Processing Center (LDPC) via wired or 4G wireless connections. The LDPC performs homologous correlation of electromagnetic pulse data collected from all detection stations, conducts three-dimensional localization calculations, matches positioning results with corresponding waveform data, and finally executes signal type labeling to generate a comprehensive LEMPs dataset.

**Figure 2:**
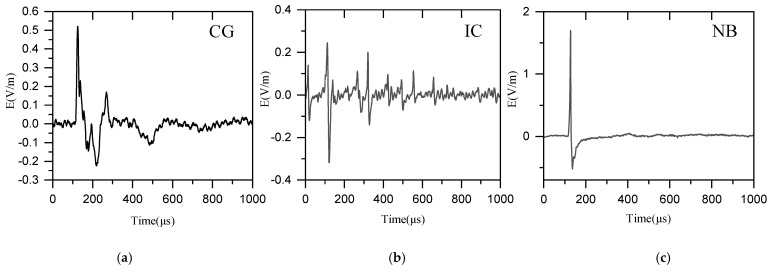
Example of some typical lightning waveforms. (**a**) cloud-to-ground flashes (CG), (**b**) ordinary intra-cloud flashes (IC), (**c**) Narrow Bipolar Events (NB).

This study categorizes the identified LEMPs into three primary types: cloud-to-ground flashes (CG), ordinary intra-cloud flashes (IC), and Narrow Bipolar Events (NB). The typical waveforms are shown in Figure 2.

**Figure 3:**
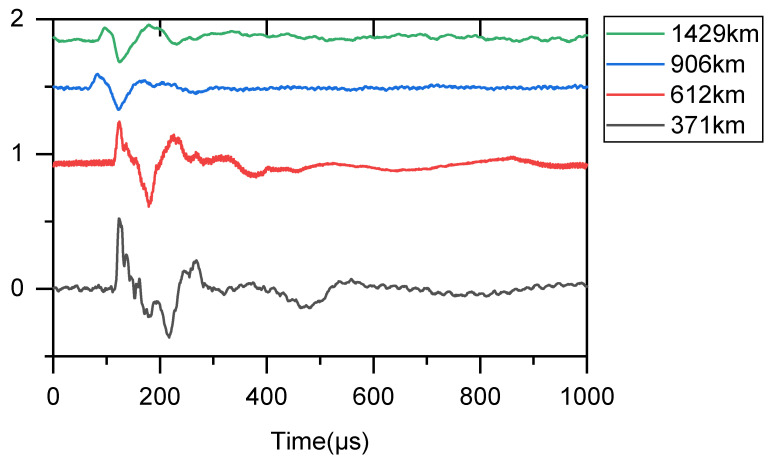
Waveforms of a Cloud-to-Ground Flash Electromagnetic Pulse Recorded by Detection Stations at Different Distances.

Figure 3 displays a cloud-to-ground flash waveform collected at 08:07:00.3333318 UTC on 15 October 2025. The black trace corresponds to the waveform received at the 371 km detection station, the red trace to the 612 km station, the blue trace to the 906 km station, and the green trace to the 1429 km station. All lightning detection stations recorded LEMPs with a duration of 1 ms. The vertical axis has no specific physical dimension.

### 2.2. Pre-Processing

As a critical preliminary step in signal analysis and modeling, data preprocessing directly affects the performance of subsequent identification models. It mainly consists of three parts:

(1) Zero-Phase Digital Filtering [19]. Considering that the main energy of VLF/LF LEMPs is concentrated in the low-frequency band, a 3rd-order Butterworth band-pass filter (zero-phase type) was adopted to process the raw signals for noise interference reduction. The filter’s passband range is set to 3–150 kHz, which is narrower than the 3–400 kHz operating band of the detection station. This selection is based on practical physical considerations: the detection station operates in field environments and is susceptible to ambient electromagnetic interference. Restricting the filter passband to the low-frequency range (3–150 kHz) can improve the signal-to-noise ratio of the target signals.
(2) Normalization. To eliminate interference from amplitude variations in raw signals and ensure data consistency across different observation conditions, all LEMPs were subjected to standardized normalization. This process employs a linear transformation to map signal amplitudes to a unified range of [−1, 1]. The normalization procedure not only mitigates the influence of signal intensity differences but also enhances numerical stability during subsequent computational processing.
(3) To meet the deep learning model’s requirement for consistent input data length, the continuous signals need to be segmented. Based on the statistical analysis of the time-domain characteristics of LEMPs, the signals were divided into data segments with a fixed length, where each segment contains 1000 sampling points. The peak point corresponds to the 120th sample among the 1000 samples, which means multiple waveforms are aligned by their peak points. This ensures that each segment completely covers the rising edge, peak value, and decay process of a single LEMP. For the residual part at the end of the signal with fewer than 1000 points, zero-padding was adopted to supplement it, so as to ensure the integrity of the data structure.

### 2.3. Homologous Signal Matching

The time window method [20] was adopted to quickly and preliminarily screen the detection data whose signal time falls within the time window from the signal queue.

By calculating the maximum distance $D_{max}$ between Lightning detection stations, the maximum time difference $W_{t}$ for an event source to propagate to each station can be derived. Since each Lightning detection station is equipped with a GPS/BeiDou positioning and timing device, their time is synchronized with the global timing system, and the time synchronization error between stations can be better than 50 ns. Therefore, the inter-station synchronization error can be neglected, and $W_{t}$ is used as the time window for homologous electromagnetic pulse matching. The calculation formula is as follows:

(1) $$W_{t}=\frac{D_{max}}{c}$$

where *c* represents the propagation velocity of electromagnetic pulses in air, typically taken as *c* = 3 × 10^8^ m/s.

For the pre-screened signals, a sliding time window is employed to calculate the cross-correlation coefficient [21] between two temporally proximate homologous pulse signals, thereby further refining the selection by eliminating non-homologous pulses (those with low cross-correlation coefficients). The cross-correlation coefficient $r_{t}$ between two electromagnetic pulse sequences $X_{i},\;Y_{i}$ is calculated as follows:

(2) $$r_{t}=\frac{\sum_{i=1}^{n}\left(X_{i}-\bar{X}\right)\left(Y_{i}-\bar{Y}\right)}{\sqrt{\sum_{i=1}^{n}{\left(X_{i}-\bar{X}\right)}^{2}}\sqrt{\sum_{i=1}^{n}{\left(Y_{i}-\bar{Y}\right)}^{2}}}$$

**Figure 4:**
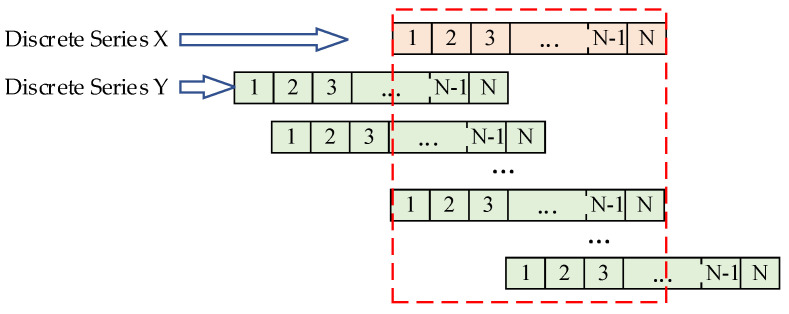
Schematic Diagram of Cross−Correlation Coefficient Calculation Using Sliding Time Window. (The red dashed box indicates the numerical range of discrete series X and Y for each calculation).

Figure 4 demonstrates the method for calculating cross-correlation coefficients between two LEMPs using a sliding window. For data sequences of length N, N cross-correlation coefficients $r_{t}$ can be computed. This approach yields more accurate correlation measurements while effectively mitigating the impact of potential signal acquisition deviations on the results.

**Figure 5:**
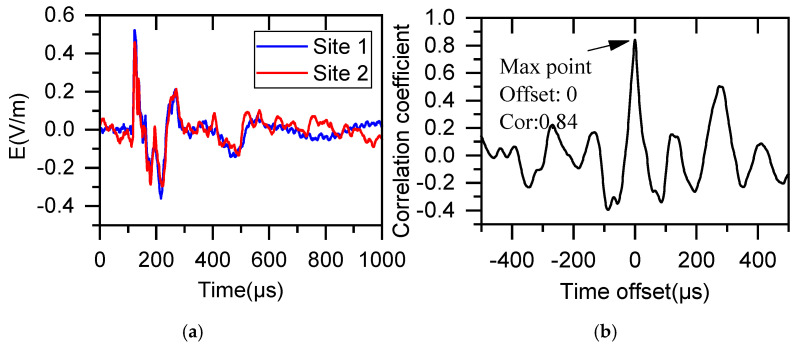
Results of sliding cross−correlation computation for two LEMPs. (**a**) Waveform of two signal sequences, (**b**) Computed cross−correlation coefficients.

Figure 5 presents the cross-correlation computation results for a set of LEMPs using a sliding window approach. The obtained correlation coefficient of 0.84 indicates a positive correlation between the two waveforms.

### 2.4. Model Development and Training

To address the requirements for lightning event localization, this study adopts a Branch-Fusion convolutional neural network (CNN) architecture. As the localization computation for individual lightning events requires observational data from at least four detection stations as input, the network structure is designed to comprise four feature extraction branches and one multi-feature fusion layer. The four feature extraction branches have identical structures, and they, respectively, perform feature extraction and dimension reduction on the raw data collected by each detection station. The multi-feature fusion layer subsequently integrates the feature vectors output from the four branches, ultimately mapping them to corresponding lightning signal categories to achieve classification.

#### 2.4.1. Feature Extraction Branch Network

**Figure 6:**
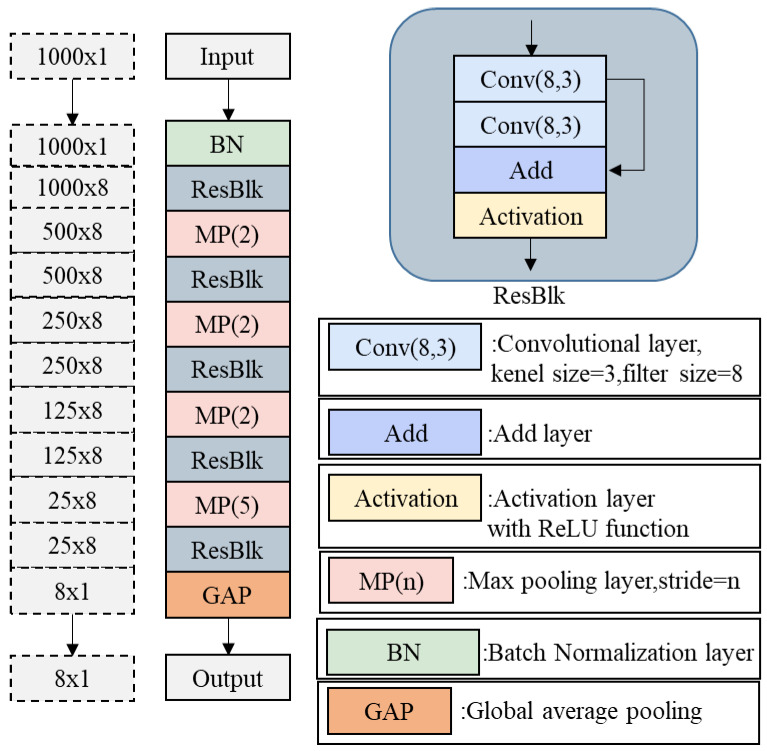
Detailed Network Architecture of a Signal Feature Extraction Branch.

The specific structure of a single feature extraction branch is shown in Figure 6. This branch is composed of an input layer, convolutional layers, batch normalization layers, activation layers, pooling layers, and residual blocks, stacked in a specific order. The parameters of the signal feature extraction network model are presented in Table 1.

These features are quantitative indicators that capture the intrinsic properties of LEMP signals, serving as condensed representations of the raw data after feature extraction and dimensionality reduction. In previous signal discrimination, easy-to-calculate parameters such as signal intensity, pulse rise time, fall time, pulse width, and signal steepness were generally used to represent the characteristics of the signal. Due to the interpretability of neural network calculations, we cannot determine whether the parameters output by the network correspond one-to-one with these parameters.

Functions and Implementation Methods of Each Layer in the Feature Extraction Branch Network:

1. Convolutional Layer

The convolutional layer is designed to autonomously learn discriminative features from input data. In this study, it specifically targets key temporal characteristics of lightning signals, including signal intensity, pulse rise time, fall time, and pulse width. The layer employs a one-dimensional convolution operation, mathematically expressed as:

(3) $$X_{j}^{l}=f(\sum_{i\in M_{j}}X_{i}^{l-1}*K_{ij}^{l}+b_{j}^{l})$$

where $X_{j}^{l}$ denotes the output feature vector of the *j*-th filter channel in the *l*-th layer. $K_{ij}^{l}$ represents the one-dimensional convolution kernel connecting the output feature vector $X_{i}^{l-1}$ from the previous layer to the current feature vector $X_{j}^{l}$, with the kernel size set to 1 × 3. The symbol * denotes the convolution operator. $b_{j}^{l}$ indicates the bias term between the feature result $X_{i}^{l-1}$ from the previous layer and the current feature result $X_{j}^{l}$. $M_{j}$ denotes the set of feature maps from the previous layer connected to $X_{i}^{l-1}$. $f\left(\cdot \right)$ represents the nonlinear activation function. The subscript i denotes the *i*-th feature point in the previous layer’s output, while j denotes the *j*-th feature point in the current layer. The superscript l indicates the index of the current layer. Specifically, when *i* = 1 and *l* = 1, $X_{1}^{0}$ corresponds to the input data of the network.

2. Batch Normalization Layer and Activation Layer

The feature vectors generated by the convolutional layer are first processed through a batch normalization layer. This layer implements the Batch Normalization (BN) algorithm, which standardizes the feature vectors to stabilize the gradient distribution during training, accelerate network convergence, and alleviate the vanishing gradient problem.

The output of the batch normalization layer is subsequently fed into the activation layer. This study employs the Rectified Linear Unit (ReLU) as the activation function. ReLU introduces nonlinear mapping capabilities to the neural network, enabling it to approximate arbitrary nonlinear functions. The mathematical expression of the ReLU function is defined as:

(4) $$f\left(x\right)=max(x,0)$$

where x denotes the output feature value from the batch normalization layer.

3. Pooling Layer

The pooling layer comprises a Max Pooling layer and a Global Average Pooling (GAP) layer. Both types of pooling layers primarily serve to reduce data dimensionality, enhance feature robustness, and lower the computational complexity of the network. Specifically, the GAP layer compresses each feature vector into a single feature value, effectively suppressing network overfitting through regularization without requiring additional learnable parameters. The mathematical expression for the max pooling operation is defined as:

(5) $$X_{j}^{l}(n)=\underset{r\in R}{\max}\left[X_{i}^{l-1}(n\times S+r)\right]$$

The mathematical expression for the GAP layer is defined as:

(6) $$X_{j}^{l}(n)=\underset{r\in R}{\mathrm{mean}}\left[X_{i}^{l-1}(n\times S+r)\right]$$

where $X_{j}^{l}(n)$ denotes the *n*-th output feature value of the *j*-th channel in the *l*-th layer, R represents the index set of elements within the pooling window, r indicates the local index of elements in the pooling window, S denotes the stride of the pooling operation, and n signifies the index of the *n*-th feature value in the output result.

4. Residual Blocks

The residual block is designed based on the Residual Network (ResNet) architecture. Each residual block contains two convolutional layers, each followed by BN and ReLU operations, and ends with a max pooling layer. By incorporating an identity mapping mechanism, this structure effectively mitigates issues of vanishing/exploding gradients and network degradation during deep network training, ensuring sustained feature extraction capability as network depth increases.

Meanwhile, the residual blocks facilitate cross-layer integration of features from different network levels, leveraging the fusion of multi-level features to extract enhanced feature representations with improved correlation. The parameters of residual blocks in the signal feature extraction network are shown in Table 2.

After processing through the aforementioned layers, the output of a single feature extraction branch is a feature vector of dimensions 1 × 8, which encapsulates the essential information from individual monitoring station observations through 8 key feature values.

#### 2.4.2. Multi-Feature Fusion Network

**Figure 7:**
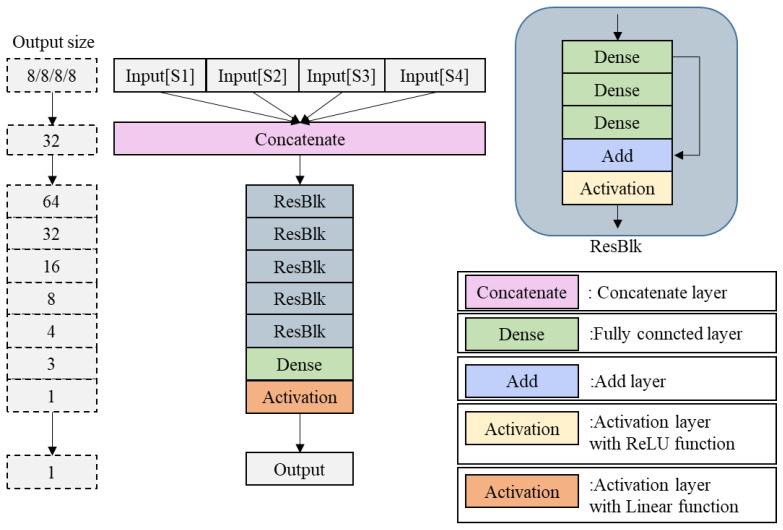
Architecture Diagram of Multi-feature Fusion Network.

The primary function of the multi-feature fusion network is to integrate the local features extracted from the four detection station branches, formulate a cross-station global feature representation, and establish the mapping relationship between these features and lightning signal identification. As illustrated in Figure 7, the network is constructed through sequential stacking of an input layer, a concatenation layer, residual blocks, fully connected layers, an activation layer, and an output layer. The parameters of the Multi-Feature fusion network model are presented in Table 3.

5. Input Layer and Concatenation Layer

The input layer of the fusion network receives the outputs from the four feature extraction branches, specifically the 1 × 8 feature vectors corresponding to each detection station. These feature vectors are denoted as $D_{S1}$ (Station 1 features), $D_{S2}$ (Station 2 features), $D_{S3}$ (Station 3 features), and $D_{S4}$ (Station 4 features). The concatenation layer restructures these four input features through a feature concatenation strategy to form a global feature vector *D*, expressed as:

(7) $$D=\left[D_{S1},D_{S2},D_{S3},D_{S4}\right]$$

Since each input feature vector has a dimension of 1 × 8, the concatenated global feature vector D attains a dimension of 1 × 32 (i.e., 8 + 8 + 8 + 8 = 32), thereby achieving complete integration of the feature information from all four stations.

6. Feature Fitting Module (Residual Blocks + Fully Connected Layers)

The 1 × 32 global feature vector output from the concatenation layer is subsequently fed into a feature recognition module composed of residual blocks and fully connected layers. The residual blocks maintain the same architectural design as described in Section 2.4.1, based on the ResNet framework, to mitigate the vanishing gradient problem during deep-level fitting and ensure effective propagation of feature information. The fully connected layers then transform the high-dimensional comprehensive features into low-dimensional correlated features through weight matrices, establishing the foundation for subsequent predictions in the output layer. The parameters of the residual blocks in the Multi-Feature fusion network are presented in Table 4.

7. Output Layer

The final layer of the model is the output layer, which utilizes a 3 × 1 fully connected layer combined with a Softmax activation function to yield the data type identification results. Thus, the output layer computation can be expressed as follows:

(8) $$Y_{j}=\frac{e^{X_{j}}}{\sum_{j=1}^{3}e^{X_{j}}}$$

where $X_{j}$ represents the *j*-th feature value of the *X*-th layer, and $Y_{j}$ represents the *j*-th feature value of the *Y*-th layer. The output is the lightning type identification and classification result, i.e., CG, IC, or NB.

## 3. Results

To evaluate the model performance, 50 independent training experiments were conducted, each employing different random seeds to ensure reproducibility of the results. During each training session, classification accuracy was monitored simultaneously on both the training and test sets to prevent overfitting.

### 3.1. Dataset Characteristics

**Figure 8:**
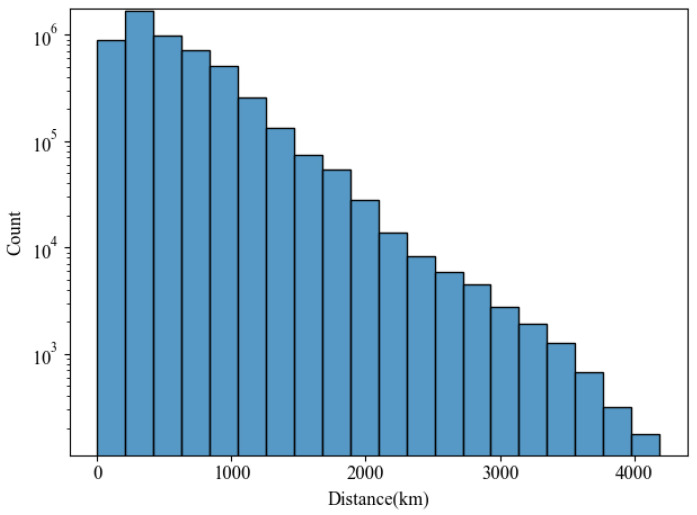
Distance Distribution of Lightning Event Data.

This study established three distinct LEMP datasets: cloud-to-ground flashes (CG), ordinary intra-cloud flashes (IC), and Narrow Bipolar Events (NB). Each category contains 100,000 samples, resulting in a total of 300,000 samples. The labeled dataset was randomly partitioned into training and testing sets, with the training set comprising 70% of the total data and the remainder allocated for testing. The training set was utilized for model parameter training and learning, while the testing set served to evaluate the model’s learning efficacy and identification performance. All data are derived from the measurements of a high-precision ADTD lightning detection network and have undergone manual analysis and verification, ensuring the type accuracy of the training and test data. The distance-dependent distribution of lightning events in the dataset is illustrated in Figure 8. The horizontal axis represents the distance between the lightning detection station and the location where the lightning occurs, and the vertical axis represents the frequency of lightning samples used for the model.

The sample data exhibits a pronounced decreasing trend with increasing distance. Within the proximal range (0–1000 km), the sample count reaches its maximum, on the order of 10^5^ to 10^6^. Beyond 1000 km, the number of samples declines rapidly, dropping below 10^2^ at 4000 km. A substantial disparity in sample size exists across different distance intervals. The sample count in the proximal range (e.g., 0–500 km) exceeds that of long-range intervals (e.g., 3000–4000 km) by three to four orders of magnitude. This pronounced imbalance in distribution is primarily attributed to the convenience and abundance of empirical data collection in proximal regions.

### 3.2. Model Performance

**Figure 9:**
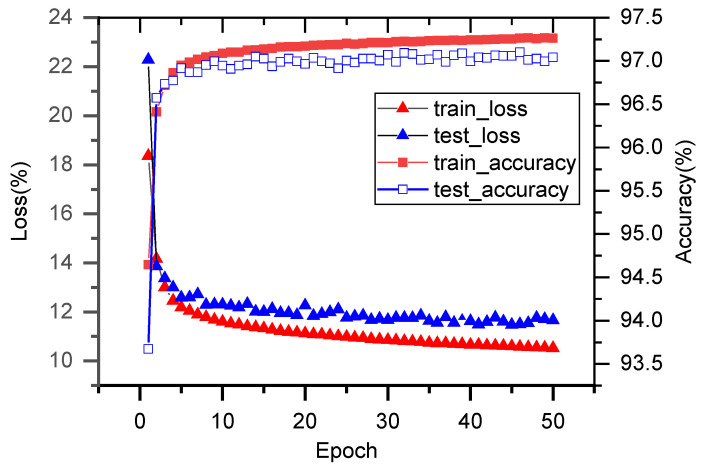
Model training and testing results.

Experimental results demonstrate that the model achieved a high identification accuracy of 97.26% on the training set and 97.10% on the test set. These results indicate that the proposed Branch-Fusion CNN possesses strong learning capability and excellent generalization performance in LEMPs classification tasks (as shown in Figure 9). Analysis of the training and validation trends reveals that the model rapidly converged during the initial phase and maintained stable performance throughout subsequent training iterations, demonstrating its excellent optimization characteristics.

Furthermore, the repeated experiments with 50 independent training trials further validate the model’s stability. Each training session consistently achieved high accuracy with minimal performance fluctuations, demonstrating the method’s strong robustness and reliability (see Figure 9). This indicates that the model maintains consistently high performance even under different random initialization conditions, providing solid technical assurance for practical applications.

### 3.3. Distance-Dependent Accuracy

**Figure 10:**
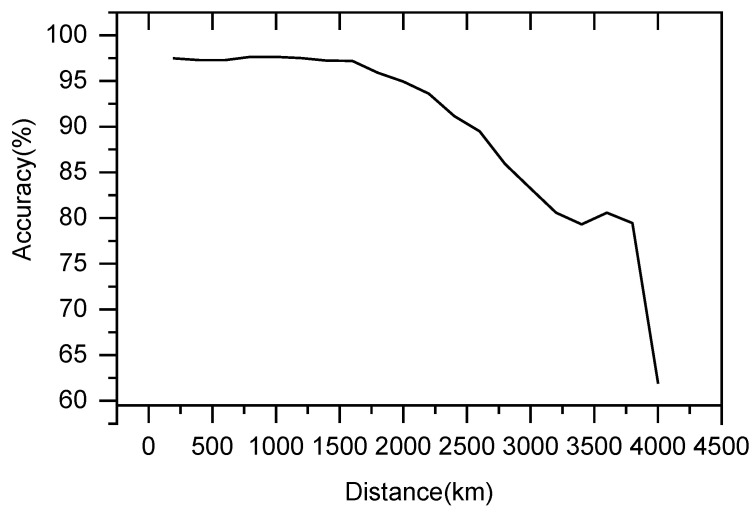
Distance-Dependent LEMPs Identification Accuracy.

Simultaneously, we investigated the identification efficiency of lightning at different distances using the proposed model. As shown in Figure 10, the model’s identification accuracy demonstrates significant distance dependency, which can be characterized by three distinct phases:

1. High-Stability Phase at Close Range (0–1500 km)

Within the 0–1500 km range, the model maintains a consistently high identification accuracy above 95%, with the performance curve remaining nearly flat. This behavior is closely associated with the preservation of signal characteristics during short-range electromagnetic pulse propagation: at close distances, signal attenuation is minimal, and temporal features (such as pulse rise time and width) remain intact. The feature extraction branch network can fully capture discriminative patterns, while the multi-feature fusion network achieves precise classification based on high signal-to-noise ratio characteristics. Consequently, the model demonstrates exceptional stability and recognition capability in this range.

2. Moderate-Distance Attenuation Phase (1500–3500 km)

Beyond 1500 km, the identification accuracy exhibits a gradual linear decline, decreasing from over 95% to approximately 80%. This performance degradation primarily stems from signal attenuation during long-distance propagation: as distance increases, signal strength diminishes, resulting in lower signal-to-noise ratios of features captured by the extraction branches. Additionally, factors such as inter-station synchronization errors and propagation path discrepancies negatively impact the precision of feature fusion, collectively contributing to the observed decline in recognition accuracy.

3. Long-Distance Degradation Phase (Beyond 3500 km)

Beyond 3500 km, the model’s accuracy drops precipitously, declining sharply from around 80% to below 60%. This drastic deterioration is fundamentally attributed to the low accuracy of feature estimates under extreme-distance conditions: after long-range propagation, temporal characteristics of signals become severely distorted, and their intensity approaches noise-level thresholds, making it challenging for feature extraction branches to capture discriminative patterns. Furthermore, the location accuracy in multi-station collaborative localization increases significantly at such distances, further reducing the feature utilization efficiency of the fusion network, ultimately leading to a collapse in model performance.

## 4. Discussion

This study achieves efficient classification of LEMPs under multi-station detection by employing a Branch-Fusion CNN, demonstrating outstanding performance. The model achieves identification accuracies of 97.26% on the training set and 97.10% on the test set, respectively. Furthermore, 50 independent training experiments validate its strong learning capability, excellent generalization performance, as well as high robustness and stability. These results can be attributed to the following key factors:

First, the multi-branch architecture demonstrates significant design advantages. This framework enables parallel processing of signals from different detection stations, effectively capturing unique characteristics from each site. Subsequently, the multi-feature fusion network integrates cross-station information, substantially improving classification accuracy and adaptability to multi-station scenarios. Compared to existing single-station or single-branch processing methods, this parallel-fusion paradigm fully leverages the spatial information advantages of multi-station detection, providing richer feature dimensions for classifying complex electromagnetic pulse signals.

Second, the feature extraction capability of Branch-Fusion CNN serves as the core foundation for the model’s performance. It enables in-depth mining of underlying characteristics in lightning electromagnetic pulses, including nonlinear variation patterns in weak signals. These features are crucial for distinguishing between different types of LEMPs.

As presented in Table 5, the proposed Branch-Fusion CNN achieves a lightning identification and classification accuracy of 97.10% on 300,000 samples, slightly outperforming Zhu et al.’s Random Forest (97%) [22] and Leal et al.’s ResNet (96.97%) [10], and significantly surpassing existing methods of SVM (95%) [22], CNN (90%) [23] and MRTransformer (90%) [11]. Its advantages stem from the architectural design of multi-branch parallel extraction of multi-station signal features and a fusion module that filters noise from individual branches. However, the Branch-Fusion CNN developed in this work, trained on large-scale empirical data covering diverse scenarios, encompasses a broader feature space of signal characteristics, thereby demonstrating superior adaptability to complex real-world environments.

To address the issues of signal synchronization and noise suppression in multi-station detection systems, this study employs a 3rd-order Butterworth zero-phase band-pass filter (3–150 kHz) to mitigate ambient electromagnetic interference while preserving critical LEMP signal characteristics. Precise signal synchronization is achieved through homologous signal matching based on the unique time-domain and waveform features of LEMP signals. To resolve the insufficient discriminability of LEMP signals in highly complex electromagnetic environments, a novel Branch-Fusion CNN architecture is developed, which enables the effective extraction and adaptive integration of complementary feature representations from multi-station signals, thereby enhancing the distinguishability of different LEMP types. Regarding the inadequate robustness of algorithms in practical field applications, the multi-branch parallel processing framework reduces the model’s sensitivity to data perturbations (e.g., signal attenuation, local noise interference), while the dedicated fusion mechanism filters out noise-induced errors from individual branches, improving the stability and reliability of the entire identification system.

However, this study has several limitations. First, the model’s sensitivity to hyperparameters (e.g., learning rate, network depth, and optimization algorithms) may affect its consistent performance across different application scenarios. Second, although the test set accuracy reaches 97.10%, a slight gap remains compared to the training set, suggesting potential limitations in the model’s generalization capability when encountering unseen data. Furthermore, the results indicate a significant distance-dependent performance degradation. Within the close-range interval (0–1500 km), the model maintains high identification accuracy above 95%, attributable to well-preserved LEMP features and high signal-to-noise ratios (SNR) at short distances. Under these conditions, the multi-branch network effectively captures discriminative features from each station, while the fusion network achieves precise classification based on high-fidelity characteristics. In the medium-range interval (1500–3500 km), identification accuracy exhibits a gradual linear decline. This attenuation primarily stems from signal propagation loss over long distances, where weakened signal strength and enhanced noise interference reduce the SNR of features extracted by the branches. This observation suggests that for medium-range applications, performance improvements should focus on signal enhancement strategies, such as adaptive noise suppression. Beyond 3500 km, the model’s accuracy drops drastically due to “feature degradation” under extreme-distance conditions: after long-range propagation, temporal features become severely distorted, and signal intensity approaches noise levels, making it challenging for the multi-branch network to extract discriminative patterns. This sharp performance decline highlights the current model’s limitations in handling ultra-long-distance, low-SNR signals. Future work should focus on developing targeted feature compensation algorithms for distant signals or optimizing multi-station deployment to overcome this performance bottleneck.

Several directions warrant further investigation. First, the introduction of automated hyperparameter optimization algorithms (e.g., Bayesian optimization) could enhance the model’s adaptability and stability. Second, for application scenarios with stringent real-time requirements, lightweight network architectures or edge computing technologies should be explored to improve processing efficiency. Furthermore, increasing the number of long-distance signal samples, developing adaptive noise suppression techniques, and designing feature compensation algorithms for distant signals would significantly enhance long-range identification performance.

In summary, this study proposes an efficient and reliable multi-station identification method for LEMPs based on deep learning, with experimental results validating its superiority. However, further optimization of both model architecture and training strategies remains necessary to address more complex practical requirements.

## 5. Conclusions

This study presents a comprehensive investigation of LEMPs classification using a multi-branch deep convolutional neural network, yielding several key findings. First, experimental results demonstrate that the proposed model achieves identification accuracies of 97.26% on the training set and 97.10% on the test set, significantly outperforming both existing methods and single-branch deep learning approaches. Second, the multi-branch architecture exhibits distinct advantages in feature extraction and classification performance by enabling parallel processing of multi-station signal information. Third, the consistent outcomes across 50 independent training experiments confirm the model’s robustness and generalization capability. These results collectively validate the efficiency and reliability of the multi-branch deep convolutional neural network for classifying complex LEMPs.

However, the identification efficiency of the proposed model exhibits significant distance-dependent performance, which is closely linked to the physical propagation characteristics of LEMPs, data distribution patterns, and the sufficiency of model training. The high accuracy at close range validates the model’s strong capability in scenarios with well-preserved signal features. The performance decline at medium distances suggests the need for optimization in signal enhancement and multi-station collaboration. The sharp accuracy drop at long distances reflects the model’s limitations under low signal-to-noise ratios and feature degradation scenarios. Future improvements should focus on long-distance feature compensation algorithms, optimized multi-station deployment, and balanced data distribution strategies to enhance model robustness in long-range scenarios.

From a scientific research perspective, this study provides a novel solution for signal classification in complex electromagnetic environments and validates the potential of deep learning methods in handling nonlinear feature extraction and high-precision classification tasks. The findings offer significant references for theoretical research and technological development in related fields.

Although this study has limitations, including model sensitivity to hyperparameters, room for improvement in generalization capability, and disproportionate representation of close-range data, future research improvements in automated parameter tuning, lightweight architectures, data sample enhancement, and physical interpretability are expected to further optimize model performance to better meet practical application requirements.

In conclusion, the multi-station identification method for LEMPs based on deep learning proposed in this study demonstrates high efficiency and reliability, providing new insights and technical support for the development of lightning electromagnetic pulse identification technology, with substantial theoretical significance and practical application value.

## Figures and Tables

**Table 1:** Parameter of a signal feature extraction network model.

No.	Layer Name	Filter Number	Kernel Size	Pooling Size	Padding	Stride	Activation Function	Output Shape
1	Input	/	/	/	/	/	/	(1000, 1)
2	Batch Normalization	/	/	/	/	/	/	(1000, 1)
3	Residual Block	/	/	/	/	/	/	(1000, 8)
4	Max-Pooling	/	/	2	/	2	/	(500, 8)
5	Residual Block	/	/	/	/	/	/	(500, 8)
6	Max-Pooling	/	/	2	/	2	/	(250, 8)
7	Residual Block	/	/	/	/	/	/	(250, 8)
8	Max-Pooling	/	/	2	/	2	/	(125, 8)
9	Residual Block	/	/	/	/	/	/	(125, 8)
10	Max-Pooling	/	/	5	/	5	/	(25, 8)
11	Residual Block	/	/	/	/	/	/	(25, 8)
12	Global Average Pooling	/	/	25	/	/	/	(8, 1)
13	Output Layer	/	/	/	/	/	/	(8, 1)

**Table 2:** Parameters of residual blocks in the signal feature extraction network.

No.	Layer Name	Filter Number	Kernel Size	Pooling Size	Padding	Stride	Activation Function	Output Shape
1	Input	/	/	/	/	/	/	(N, 1)
2	Convolutional Layer	3	8	/	Yes	/	ReLU	(N, 8)
3	Convolutional Layer	3	8	/	Yes	/	ReLU	(N, 8)
4	Add Layer	/	/	/	/	/	/	(N, 8)
5	Activation Layer	/	/	/	/	/	ReLU	(N, 8)
6	Output Layer	/	/	/	/	/	/	(N, 8)

N denotes the length of the input data.

**Table 3:** Parameter of a multi-feature fusion network model.

No.	Layer Name	Units	Activation Function	Output Shape
1	Input-1	/	/	(8, 1)
2	Input-2	/	/	(8, 1)
3	Input-3	/	/	(8, 1)
4	Input-4	/	/	(8, 1)
5	Concatenate Layer	/	/	(32, 1)
6	Residual Block	64	/	(64, 1)
7	Residual Block	32	/	(32, 1)
8	Residual Block	16	/	(16, 1)
9	Residual Block	8	/	(8, 1)
10	Residual Block	4	/	(4, 1)
11	Dense Layer	/	/	(3, 1)
12	Activation Layer	/	Softmax	(3, 1)
13	Output Layer	/	/	(3, 1)

**Table 4:** Parameters of residual blocks in the Multi-Feature fusion network.

No.	Layer Name	Units	Activation Function	Output Shape
1	Input	M	/	(M,1)
2	Dense Layer	M	/	(M,1)
3	Dense Layer	2 * M	/	(2 * M,1)
4	Dense Layer	M	/	(M,1)
5	Add Layer	/	/	(M,1)
6	Activation Layer	/	ReLU	(M,1)
7	Output Layer	/	/	(M,1)

* M denotes the length of the input data.

**Table 5:** Accuracy of existing and our methods.

Author(s)	Method	Sample Size	Lightning Type(s) Identified	Accuracy
Mehranzamir, K. et al., 2019 [5]	DWT	200	CG	92%
Leal, A.F.R. et al., 2023 [10]	RestNet	25,901	CG, IC, CID	96.97%
Sun, J.H. et al., 2023 [11]	MRTransformer	12,000	CG, IC, PBP, NBE, MP, SW	90%
Zhu, S.X. et al., 2024 [22]	Random forest	30,000	CG, IC, NBE	97%
Zhu, S.X. et al., 2024 [22]	SVM	30,000	CG, IC, NBE	95%
Wang, C.X. et al., 2025 [23]	CNN	3300	CG, IC, Other	90%
Ours	Branch-Fusion CNN	300,000	CG, IC, NB	97.10%

## Data Availability

The raw data supporting the conclusions of this article will be made available by the authors on request.
